# Dexmedetomidine Prevents Lipopolysaccharide-Induced MicroRNA Expression in the Adult Rat Brain

**DOI:** 10.3390/ijms18091830

**Published:** 2017-08-23

**Authors:** Nadine Paeschke, Clarissa von Haefen, Stefanie Endesfelder, Marco Sifringer, Claudia D. Spies

**Affiliations:** 1 Department of Anesthesiology and Operative Intensive Care Medicine (CCM, CVK), Charité-Universitätsmedizin Berlin, Corporate Member of Freie Universität Berlin, Humboldt-Universität zu Berlin, and Berlin Institute of Health, Campus Virchow-Klinikum, Augustenburger Platz 1, 13353 Berlin, Germany; nadine.paeschke@charite.de (N.P.); clarissa.von-haefen@charite.de (C.v.H.); marcosifringer1969@gmail.com (M.S.); claudia.spies@charite.de (C.D.S.); 2 Department of Neonatology, Charité-Universitätsmedizin Berlin, Corporate Member of Freie Universität Berlin, Humboldt-Universität zu Berlin, and Berlin Institute of Health, Augustenburger Platz 1, 13353 Berlin, Germany; stefanie.endesfelder@charite.de

**Keywords:** neuroinflammation, dexmedetomidine, miRNA, hippocampus, cortex

## Abstract

During surgery or infection, peripheral inflammation can lead to neuroinflammation, which is associated with cognitive impairment, neurodegeneration, and several neurodegenerative diseases. Dexmedetomidine, an α-2-adrenoceptor agonist, is known to exert anti-inflammatory and neuroprotective properties and reduces the incidence of postoperative cognitive impairments. However, on the whole the molecular mechanisms are poorly understood. This study aims to explore whether dexmedetomidine influences microRNAs (miRNAs) in a rat model of lipopolysaccharide (LPS)-induced neuroinflammation. Adult Wistar rats were injected with 1 mg/kg LPS intraperitoneal (i.p.) in the presence or absence of 5 µg/kg dexmedetomidine. After 6 h, 24 h, and 7 days, gene expressions of interleukin 1-β (*IL1-β*), tumor necrosis factor-α (*TNF-α*), and microRNA expressions of miR 124, 132, 134, and 155 were measured in the hippocampus, cortex, and plasma. Dexmedetomidine decreased the LPS-induced neuroinflammation in the hippocampus and cortex via significant reduction of the *IL1-β* and *TNF-α* gene expressions after 24 h. Moreover, the LPS-mediated increased expressions of miR 124, 132, 134, and 155 were significantly decreased after dexmedetomidine treatment in both brain regions. In plasma, dexmedetomidine significantly reduced LPS-induced miR 155 after 6 h. Furthermore, there is evidence that miR 132 and 134 may be suitable as potential biomarkers for the detection of neuroinflammation.

## 1. Introduction

Neuroinflammation plays a major role in the pathogenesis of several neurodegenerative diseases. A clinical and experimental correlation between elevated peripheral proinflammatory cytokines and neuroinflammation has been largely described [1,2,3]. Lipopolysaccharide (LPS) or surgical trauma activate the immune system and are capable of causing memory impairment [3,4,5]. Studies indicate that high levels of the proinflammatory cytokines interleukin 1-β (*IL1-β*) and tumor necrosis factor-α (*TNF-α*) are associated with postoperative cognitive impairments [6,7]. These are characterized by neuronal dysfunction and/or neuronal death, which can induce long-lasting cognitive decline and aggravation in learning and memory [2,3,8]. Surgery, especially in the elderly, can lead to neuroinflammation, cause long-lasting postoperative cognitive impairments, and permanently affect the patient’s life [9,10]. Patients with those impairments have a higher risk of developing postoperative complications such as sepsis, and show a higher mortality rate [11,12].

Dexmedetomidine, a highly selective α-2-adrenergic agonist, exerts sedative, anxiolytic, and analgesic properties and is therefore used as perioperative sedative agent [13]. Several studies indicate that dexmedetomidine has protective properties regarding postoperative cognitive impairments in clinical trials [14,15,16,17]. Compared to other anesthetics, dexmedetomidine shortens the duration of artificial ventilation, the extubating time, and the occurrence of postoperative cognitive impairments [18,19,20,21]. Dexmedetomidine shows anti-inflammatory as well as neuroprotective properties in several cell and rodent studies [8,22,23,24,25,26,27,28,29]. In different rodent behavior experiments, dexmedetomidine also prevents the occurrence of cognitive impairments [30,31]. However, the exact molecular mechanisms of the positive and protective character of dexmedetomidine are not entirely understood.

MicroRNAs (miRNAs) are approximately 22 base pairs long, non-coding RNAs which play a significant role in post-transcriptional gene regulation. It is currently assumed that about 60% of all genes are regulated by miRNAs [32]. The majority of known miRNAs are found in the brain, whereas some of them are exclusive to neuronal tissue [33,34]. Depending on the complementarity of the binding between miRNA and mRNA, the mRNA is degraded or its translation repressed [35]. Studies have shown that the expression of certain miRNAs is altered in different neurodegenerative processes [36].

The aim of this study was to determine whether dexmedetomidine has an impact on the expression of *IL1-β*, *TNF-α*, and several miRNAs in the hippocampus and cortex in a neuroinflammation model of adult rats. Additionally, miRNAs that showed an altered expression in the brain were measured also in plasma to study whether dexmedetomidine influences LPS-induced changes and whether those miRNAs may be suitable biomarkers for the detection of neuroinflammation.

## 2. Results

### 2.1. Dexmedetomidine-Attenuated, LPS-Induced IL1-β and TNF-α Gene Expression in the Hippocampus and Cortex

**Figure 1:**
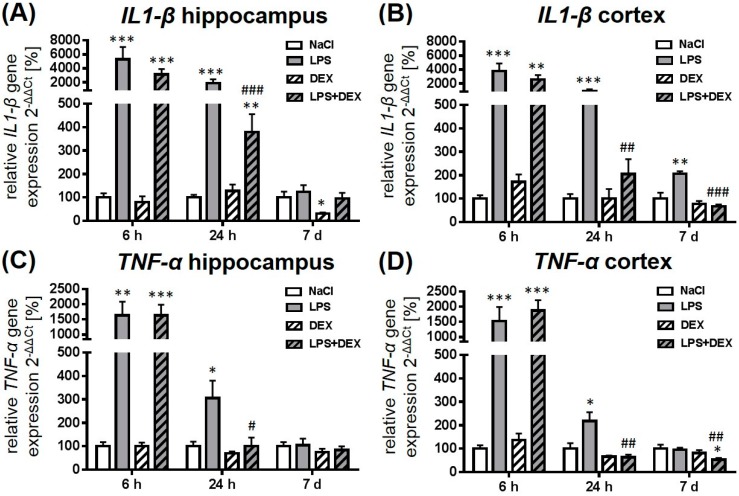
Representation of interleukin 1-β *(IL1-β)* (**A**,**B**) and tumor necrosis factor-α (*TNF-α)* (**C**,**D**) gene expression after LPS (lipopolysaccharide) and dexmedetomidine treatment. Results are shown as mean ± standard error of the mean (SEM) (*n*  =  6–8 per group). Data are normalized to levels of saline-treated rats (control  =  100%). * *p* < 0.05, ** *p* < 0.01, and *** *p* < 0.001 represent the difference compared to saline-treated groups. ^#^ *p* < 0.05, ^##^ *p* < 0.01, and ^###^ *p* < 0.001 represent the difference between LPS and LPS in combination with dexmedetomidine-treated groups.

As shown in Figure 1, the administration of 1 mg/kg LPS caused a significant upregulation of *IL1-β* (Figure 1A,B) and *TNF-α* (Figure 1C,D) gene expression after 6 and 24 h in the hippocampus and cortex compared to the saline-treated rats. Seven days post LPS injection, the *IL1-β* expression was still significantly elevated in the cortex compared to the control. Dexmedetomidine significantly reduced the LPS-induced rise of both cytokines after 24 h, indicating an anti-inflammatory effect of this drug. Despite the dexmedetomidine-mediated reduction of LPS-induced *IL1-β* expression in the hippocampus, the expression was significantly elevated compared to the saline-treated animals. Twenty-four hours after LPS injection, the dexmedetomidine-treated animals showed *TNF-α* levels similar to the control group in both brain regions. Seven days after LPS injection dexmedetomidine was still able to reduce the LPS-mediated rise of *IL1-β* in the cortex. The drug alone significantly decreased hippocampal *IL1-β* expression after 7 days.

### 2.2. Dexmedetomidine Modulated the miRNA Expression in the Hippocampus, Cortex, and Plasma of LPS Treated Rats

The aim of this study was to determine whether dexmedetomidine influences the expression of different miRNAs in the brain and the plasma of adult rats in a neuroinflammation model.

#### 2.2.1. Expression of MicroRNA 124

**Figure 2:**
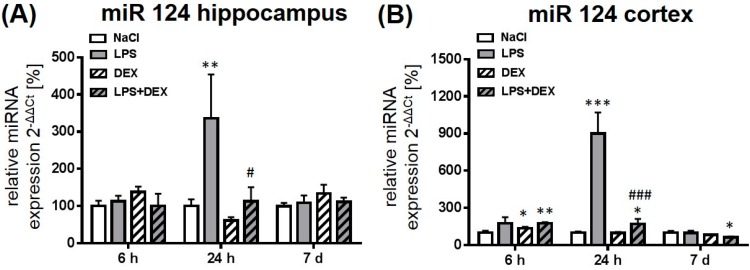
Representation of miR 124 expression after LPS and dexmedetomidine treatment in the hippocampus (**A**) and cortex (**B**). Results are shown as mean ± SEM (*n*  =  6–8 per group). Data are normalized to levels of saline-treated rats (control  =  100%). * *p* < 0.05, ** *p* < 0.01, and *** *p* < 0.001 represent the difference compared to saline-treated groups. ^#^ *p* < 0.05 and ^###^ *p* < 0.001 represent the difference between LPS and LPS in combination with dexmedetomidine-treated groups.

As shown in Figure 2, the administration of LPS mediated a significant increase of miR 124 expression after 24 h in the hippocampus (Figure 2A) and the cortex (Figure 2B) compared to the respective control groups. LPS-induced expression of miR 124 was more pronounced in the cortex than in the hippocampus. After 7 days the endotoxin had no influence on the expression of this miRNA. LPS in combination with dexmedetomidine prevented LPS-induced miR 124 expression after 24 h in both investigated brain regions significantly. Six hours after LPS and dexmedetomidine administration miR 124 was significantly enhanced in the cortex, whereas it was significantly reduced after 7 days compared to the respective control. Moreover, the drug alone caused a significant, enhanced miR 124 expression after 6 h in the cortex when compared to the control. In plasma, miR 124 expression was not detectable at any time point.

#### 2.2.2. Expression of MicroRNA 132

**Figure 3:**
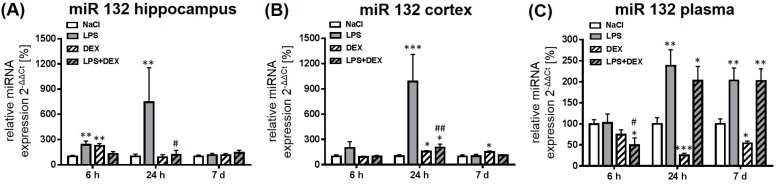
Representation of miR 132 expression after LPS and dexmedetomidine treatment in the hippocampus (**A**), cortex (**B**), and plasma (**C**). Results are shown as mean ± SEM (*n*  =  6–8 per group). Data are normalized to levels of saline-treated rats (control  =  100%). * *p* < 0.05, ** *p* < 0.01, and *** *p* < 0.001 represent the difference compared to saline-treated groups. ^#^ *p* < 0.05 and ^##^ *p* < 0.01 represent the difference between LPS and LPS in combination with dexmedetomidine-treated groups.

Rats treated with LPS showed a significant upregulation of miR 132 expression in the hippocampus after 6 and 24 h when compared to the control, whereas the endotoxin had no influence on the expression after 7 days (Figure 3A). Dexmedetomidine in combination with LPS significantly prevented the LPS-mediated upregulation of miR 132 in this brain region after 24 h. The drug alone caused a significant rise of miR 132 expression after 6 h in the hippocampus that is comparable with the LPS-induced expression.

In the cortex, treatment with LPS significantly enhanced the expression of miR 132 after 24 h, which was significantly reduced by administration of dexmedetomidine (Figure 3B). The drug alone rose miR 132 in the cortex after 24 h and 7 days in a significant way when compared to saline-treated rats.

The miR 132 expression in plasma significantly increased 24 h and 7 days post LPS administration, what was not influenced by dexmedetomidine (Figure 3C). After 6 h, the combination of dexmedetomidine and LPS significantly reduced the miR 132 expression in plasma compared to the control and the LPS group. The drug alone reduced plasma miR 132 after 24 h and 7 days significantly.

#### 2.2.3. Expression of MicroRNA 134

**Figure 4:**
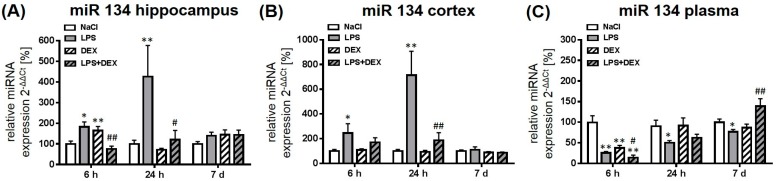
Representation of miR 134 expression after LPS and dexmedetomidine treatment in the hippocampus (**A**), cortex (**B**), and plasma (**C**). Results are shown as mean ± SEM (*n*  =  6–8 per group). Data are normalized to levels of saline-treated rats (control  =  100 %). * *p* < 0.05 and ** *p* < 0.01 represent the difference compared to saline-treated groups. ^#^ *p* < 0.05 and ^##^ *p* < 0.01 represent the difference between LPS and LPS in combination with dexmedetomidine-treated groups.

In comparison to saline-treated rats, expression of miR 134 was significantly upregulated 6 and 24 h after LPS treatment in the hippocampus of adult rats (Figure 4A). Dexmedetomidine significantly attenuated LPS-induced miR 134 expression in this brain region. The drug alone caused a significant increase of miR 134 after 6 h in the hippocampus compared to control.

In the cortex, treatment with LPS also significantly increased miR 132 expression after 6 and 24 h (Figure 4B) compared to the control, what was significantly reduced by the administration of dexmedetomidine after 24 h.

In comparison to brain tissue, plasma miR 134 significantly decreased at all time points in LPS-treated animals (Figure 4C). Dexmedetomidine alone and in combination with LPS significantly reduced the expression of miR 134 after 6 h compared to saline-treated rats. The combination of dexmedetomidine and LPS enhanced the expression of miR 134 after 7 days significantly compared to the LPS group.

#### 2.2.4. Expression of MicroRNA 155

**Figure 5:**
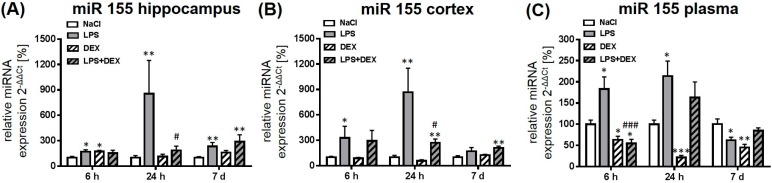
Representation of miR 155 after LPS and dexmedetomidine treatment in the hippocampus (**A**), cortex (**B**), and plasma (**C**). Results are shown as mean ± SEM (*n*  =  6–8 per group). Data are normalized to levels of saline-treated rats (control  =  100%). * *p* < 0.05, ** *p* < 0.01, and *** *p* < 0.001 represent the difference compared to saline-treated groups. ^#^ *p* < 0.05 and ^###^ *p* < 0.001 represent the difference between LPS and LPS in combination with dexmedetomidine-treated groups.

LPS significantly increased the expression of miR 155 at all investigated time points in the hippocampus (Figure 5A) compared to the saline-treated rats. Application of dexmedetomidine significantly reduced LPS-mediated, enhanced miR 155 expression after 24 h, whereas it had no influence on the expression after 6 h and 7 days. The drug alone led to a significant increase of miR 155 after 6 h in the hippocampus that is comparable with the LPS-induced expression of this miRNA.

When compared to respective control groups, miR 155 was significantly enhanced in the cortex 6 and 24 h following LPS exposition (Figure 5B). Dexmedetomidine significantly inhibited the LPS-induced expression after 24 h but compared to control group the expression was still significantly increased. LPS, in combination with dexmedetomidine, significantly elevated miR 155 after 7 days in the cortex compared to the control.

In the plasma, LPS mediated a significant enhancement of miR 155 after 6 and 24 h and a significant reduction after 7 days (Figure 5C). Dexmedetomidine, in combination with LPS, significantly decreased the LPS-induced miR 155 level after 6 h in the plasma in a way that it was even significantly downregulated compared to the saline-treated animals. Moreover, the drug alone caused a decrease of miR 155 at all time points in the plasma when compared to the control animals.

## 3. Discussion

The aim of this study was to investigate the effect of dexmedetomidine on LPS-induced neuroinflammation, and miRNA expression in the hippocampus and cortex of the adult rat brain, as those brain regions play a major role in cognition and memory. Our study demonstrates that treatment with dexmedetomidine reduced LPS-mediated neuroinflammation and prevented LPS-induced enhancement of miR 124, 132, 134, and 155.

Many studies have already shown in rodents that systemic administration of LPS activates the innate immune system, which leads to neuroinflammation, memory impairment, and neuronal cell death, particularly in the cortical and hippocampal regions of the rodent brain [1,2,3,4]. High concentrations of *IL1-β* and *TNF-α* in the brain are associated with cognitive impairments [5,6,7]. Dexmedetomidine, a selective α-2-agonist, reduces the occurrence of cognitive impairments in animal and human studies, but the molecular mechanisms are not entirely understood. In the work at hand, dexmedetomidine was applied shortly before LPS treatment. The drug is known to exert protective properties when applied before intervention in several animal studies [23,30,31]. A study from Yamanaka showed that only early dexmedetomidine (applied directly after intervention), but not late dexmedetomidine (applied 24 h after intervention), exerts protective effects [31]. Some studies also show protective properties after drug treatment 30 min post-damage induction [37,38]. Nevertheless, we chose a dexmedetomidine treatment prior to LPS application to reduce potential LPS-induced damage as fast as possible. Dexmedetomidine treatment decreased LPS-induced *IL1-β* and *TNF-α* mRNA expression in the hippocampus and cortex after 24 h in our model. At this point, the *TNF-α* concentration returned to control levels, whereas *IL1-β* was still elevated in both brain regions when compared to the control. Via reducing LPS-induced *IL1-β* expression after 7 days in the cortex, dexmedetomidine seems to have long-lasting anti-inflammatory properties. Ning et al. previously published that dexmedetomidine reduced *IL1-β*, *TNF-α*, and oxidative stress in mice brain after systemic LPS administration [8]. In the lungs of septic rats, dexmedetomidine inhibits inflammation via the toll like receptor 4/myeloid differentiation factor 88/ nuclear factor kappa-light-chain-enhancer of activated B cells (TLR4/MyD88/NF-κB) signaling pathway [39]. Moreover, an involvement of the cholinergic anti-inflammatory pathway is described in dexmedetomidine´s anti-inflammatory action in different rodent studies [22,23,40].

miRNAs regulate about 60% of genes on the posttranscriptional level and play an important role in different cell processes [32]. In our work, we investigated four miRNAs that are believed to play a role in inflammation and cognition: miR 124, 132, 134, and 155. A study from 2013 proposed members of the miR-132 and miR-134 families as biomarkers for the detection of mild cognitive impairment (MCI) [41]. As the anti-inflammatory cholinergic system plays an important role in the inflammation response, we investigated miR-124 and 132, because these miRNAs are known to influence this pathway [42]. Moreover, serum expression of miRNA-155 was shown by multiple logistic regression analysis to be an independent predictive indicator for postoperative cognitive impairment after surgery [43].

LPS administration increased the expression of all investigated miRNAs at several time points significantly in the hippocampus and cortex of adult rats. The measured miRNA level was higher in the cortex than in the hippocampus, indicating a stronger involvement of the cortex in the LPS-mediated miRNA machinery. After 7 days miR 124, 132, and 134 expressions were restored to a normal level, whereas miR 155 was still significantly upregulated in the hippocampus. Expression of miR 155 is known to be high in the central nervous system (CNS) of amyotrophic lateral sclerosis (ALS) and multiple sclerosis (MS) patients, two inflammation-related disorders [44]. High expressions of miR 155 could be a potential trigger for long lasting CNS changes in our model after administration of 1 mg/kg LPS, but this hypothesis needs further research. Even if 6 h following LPS administration some miRNAs were slightly elevated in the brain, we suggest that the high cytokine expressions after 6 h are inducing the miRNAs after 24 h. It is described that cytokines induce individual miRNAs. A study from Liu et al. indicates that in LPS-stimulated alveolar macrophages, miR 132 is upregulated after measurement of high concentrations of *IL1-β* and *TNF-α* [45]. In a human monocyte cell line, miR 132 and 155 are upregulated after *IL1-β* or *TNF-α* exposure [46]. Moreover, murine macrophages exposed to *TNF-α* show an increased miR 155 expression [47]. We suspect that the level of proinflammatory cytokines is crucial for the activation of miRNAs in our model. Induction of miRNAs after LPS treatment can be explained as a potential counter reaction and/or protection against LPS-induced damage to participate in attenuating inflammation via indirect suppression of cytokines.

miR 124 is the most abundant miRNA expressed in the CNS and is an essential factor for neuronal differentiation. It is described as “neurimmiR”, indicating an involvement in the nervous and immune systems [42]. In our model, LPS treatment significantly increased miR 124 expression after 24 h in the hippocampus and the cortex. An upregulation of miR 124 could also be detected in LPS-treated mouse macrophages and mice, where it was reported to be a critical mediator of the cholinergic anti-inflammatory action [48,49].

miR 132, that is, enhanced after LPS treatment in our model in the hippocampus and the cortex, also acts as “neurimmiR” [42]. This miRNA is induced in primary human macrophages, rat alveolar macrophages, and several organs of mice after LPS treatment [45,50]. miR 132 functions as a negative regulator of the inflammatory response in alveolar macrophages by potentiating the cholinergic anti-inflammatory pathway [45]. Acetylcholinesterase, an enzyme that cleaves acetylcholine in choline and acetic acid, is a validated target of miR 132 [45,50,51]. Inhibition of this esterase leads to higher acetylcholine amounts, which can mediate anti-inflammatory properties via binding on α-7-nAChR on macrophages. Moderate overexpression of miR 132 was shown to improve cognition, whereas high overexpression of this miRNA in rat cortex and mouse forebrain is associated with cognitive impairments [52,53,54]. Moreover, miR 132 knockout animals exert deficits in memory [55]. Therefore, miR 132 expression needs to be precisely controlled in brain tissue.

miR 134 was significantly upregulated after LPS treatment in the hippocampus and the cortex in our model. This brain-specific miRNA is activity-regulated and associated with the control of dendritic spine morphology. Elevated miR 134 levels are linked to impaired synaptic plasticity [56,57], whereas overexpression of this miRNA is associated with a reduced spine volume and reduced synaptic strength. Gao et al. proved that high miR 134 expressions result in impaired long-term potentiation in the hippocampus [57].

In our study, LPS elevates the expression of miR 155 in the hippocampus the and cortex, which is in agreement with other reports. miR 155 is one of the best-described miRNAs concerning inflammation. This miRNA is described to participate in pro- and anti-inflammatory mechanisms. Quinn et al. published that LPS-induced expression of transcription factor E26 transformation-specific 2 (Ets2) is responsible for induction of miR 155 expression, but also the NF-κB and mitogen-activated protein kinase (MAPK) pathway, which is activated during LPS infection, stimulates the expression of miR 155 [58]. miR 155 can bind to several modulators of toll like receptor (TLR)/IL-1 signaling to attenuate expression of cytokines in mouse monocyte-derived dendritic cells, macrophages, and mice [59,60]. Via targeting phosphatidylinositol-3,4,5-trisphosphate 5-phosphatase 1 (SHIP1), a negative regulator of TNF-α, and suppressor of cytokine signaling 1 (SOCS1), a negative regulator of cytokines, miR 155 also mediates an increased inflammatory response. Treating mice with LPS decreases neurogenesis in the hippocampus, which might be a consequence of miR 155-mediated IL-6 production in microglia [44].

The investigated miRNAs were not just only measured in brain, but also in plasma in our study. Because of their good stability and tissue specificity, these small non-coding RNAs are suitable as circulating biomarkers. The usage of miRNAs as biomarkers has several advantages compared to other biomarkers: miRNAs are stably expressed in plasma and even low expressions can be detected by qPCR (semiquantitative Real Time PCR) [61]. Moreover, miRNAs do not have any posttranslational modifications, which can influence their measurement [61]. Identifying miRNAs as suitable biomarkers for the fast and reliable detection of neuroinflammation is important, for instance, for the fast recognition of postoperative cognitive impairments after surgery, since rapid recognition and initiation of a proper countermeasure is essential to improving the outcome of patients. In our study, plasma miR 132 increased significantly 24 h and 7 days after LPS stimulation. A study from 2015 suggests miR 132 as a circulating biomarker for the detection of MCI, a cognitive decline that is associated with a high risk of developing Alzheimer Dementia [62]. Our study supports that miR 132 might be a potential marker for the detection of neuroinflammation, because it is enhanced in plasma after massive upregulation in the brain, indicating a release of miR 132 from the brain into the plasma. To prove if miR 132 is a suitable target for the detection of neuroinflammation in plasma, further research will be necessary. Expression of miR 134 was significantly reduced at all time points after LPS treatment in our study. Avansini et al. recently published that miR 134 is downregulated in the plasma of patients with mesial temporal lobe epilepsy, whereas it is upregulated in the hippocampus of the patients [63]. Therefore, miR 134 might be a potential circulating biomarker for the detection of functional changes in the brain. Because of its downregulation after 6 h, 24 h, and 7 days, miR 134 may be used as an early and late marker of neuroinflammation. This point needs to be further investigated and validated. LPS-induced enhancement of miR 155 expression in plasma in the study at hand is, in our opinion, more a consequence of peripheral inflammation rather than an indicator of neuroinflammation, as miR 155 is unspecific and upregulated during inflammation.

To our knowledge, this study explores for the first time that dexmedetomidine significantly suppressed the expression of miR 124, 132, 134, and 155 after LPS-induced neuroinflammation in the hippocampus and cortex. Dexmedetomidine mediated suppression of the cytokines *IL1-β* and *TNF-α* may prevent massive upregulation of all investigated miRNAs, because of the lower level of inflammation, for which there is no trigger for miRNA induction. Application of the drug alone does not cause any effects, as there is no trigger to counteract any intervention. Via suppressing the LPS-induced TLR4/MyD88/NF-κB and MAPK pathway, dexmedetomidine may avert upregulation of miR 155, as NF-κB and MAPK are known stimulators of this miRNA [58]. Whether dexmedetomidine’s attenuation of miRNA expression is directly regulated in the brain or a consequence of reduced peripheral inflammation cannot be determined. This point must be further investigated, but we hypothesize that dexmedetomidine-induced anti-inflammatory peripheral actions probably reduce vagus nerve signaling to the brain, which results in reduced neuroinflammation and therefore reduced miRNA expression. Ning et al. published that LPS-induced disturbance of the blood-brain barrier can be attenuated by dexmedetomidine [8]. We speculate that this might be a consequence of the dexmedetomidine-mediated reduction of miR 155, as miR 155 is described as a negative regulator of the blood-brain barrier during neuroinflammation. As the drug partly triggers the same pathways like LPS-induced miRNAs in our model, dexmedetomidine in parts mimics the endogen miRNA function to attenuate inflammation without negative side effects. Because high amounts of miR 132 and 134 are known to participate in impaired memory function and impaired synaptic plasticity, we speculate that dexmedetomidine-mediated preventive enhancement of those miRNAs can be a potential reason for the better outcome of animals and patients profiting from dexmedetomidine in different cognition studies.

Dexmedetomidine is more likely to influence the miRNA expression in the brain than in plasma after LPS administration. In plasma, the drug downregulated miR 132, 134, and 155, in combination with LPS after 6 h compared to the control and LPS-treated groups, whereas miR 155 was the only LPS-induced miRNA in plasma. Reduction of miR 155 after 6 h can be interpreted as a sign of reduced peripheral inflammation. After 24 h and 7 days, dexmedetomidine had no influence on the LPS-induced miR 132 and 155 expressions in plasma. miR 132 and 134 are both significantly enhanced 7 days after dexmedetomidine administration in combination with LPS, indicating long-lasting miRNA changes in plasma after endotoxin in combination with drug administration.

There are several limitations in our study. First, we only measured cytokine expressions on an mRNA level, to prove our model. Second, no behavior tests were performed, so we do not know whether high concentrations of miR 132 and 134 in our model are associated with cognitive impairments and whether dexmedetomidine can reduce these. Further research is needed to clarify how dexmedetomidine exactly influences the miRNA expression to get a full understanding of its protective properties. Therefore, analyses with specific miRNA mimics and inhibitors should be performed in cell culture experiments. Moreover, dexmedetomidine was applied only once before LPS treatment, while in the clinical setting it is infused over a longer period. To prove the usage of miR 132 and 134 as potential biomarkers, a validation step is essential. This should be performed in the plasma of patients suffering from neuroinflammation.

Taken together, our data indicate for the first time that dexmedetomidine does not just only reduce LPS-mediated, enhanced cytokine expression, but also prevents LPS-induced expression of miR 124, 123, 134, and 155 in the adult hippocampus and cortex during neuroinflammation. That opens new approaches to understand the molecular mechanism of dexmedetomidine-mediated protection. Moreover, there are hints that miR 132 and 134 may be suitable plasma biomarkers for neuroinflammation.

## 4. Materials and Methods

### 4.1. Animal Model

Adult male Wistar rats (250–300 g) underwent intraperitoneal (i.p.) LPS injection in the presence or absence of the α-2-receptor agonist dexmedetomidine (DEX). Rats were treated with i.p. LPS (1 mg/kg body weight), i.p. DEX (dexdor^®^, Orion Pharma, Espoo, Finland; 5 µg/kg body weight), or control vehicle NaCl (0.9%) after a short anesthesia in isoflurane-oxygen narcosis. Animals were divided into four groups: (1) NaCl, (2) LPS, (3) DEX, and (4) LPS+DEX. DEX was administrated 10 min before LPS treatment. Rats were hosted in groups at room temperature (22 ± 2 °C) under a standard 12–12 h light-dark cycle. Food and water were available ad libitum. All animal experiments were approved and performed in accordance with the guidelines of the Charité-Universitätsmedizin Berlin, Germany and the national ethic principles (registration no. G 0145/13, 1 July 2013).

### 4.2. Tissue Preparation

After 6 h, 24 h, 7 days, the animals were sacrificed in deep isoflurane-oxygen narcosis. The rats were transcardially perfused with normal saline (pH 7.4) and then decapitated. The brain was immediately removed and divided into two hemispheres. The whole cortical and hippocampal tissue was microdissected with a stereo magnifying glass from one hemisphere and directly snap frozen in liquid nitrogen. To obtain plasma, EDTA whole blood was centrifuged at 2500× *g* for 10 min. Afterward, the plasma was centrifuged again at 1000× *g* for 15 min and stored at −80 °C.

### 4.3. RNA Extraction and Semiquantitative Real Time PCR

Total RNA containing miRNA was isolated from snap frozen hippocampus and cortex by acidic phenol/chloroform extraction (peqGOLD RNAPure; PEQLAB Biotechnologie, Erlangen, Germany) according to the manufacturer’s instructions. RNAPure FL (PEQLAB) was used for the isolation of miRNA from plasma. The RNA-precipitation from plasma was performed overnight using glycogen as a carrier.

For the mRNA analyses, 2 µg of RNA underwent DNase treatment (Ambion, Austin, TX, USA) and were reverse transcribed with 2 µM oligo d(T) 16 primer (Promega, Mannheim, Germany) and 200 U M-MLV reverse transcriptase (Promega) at 42 °C. The cDNA was quantified in real time with dye-labeled probes and primers (metabion, Planegg/Steinkirchen, Germany) (sequences shown in Table 1). Glyceraldehyde 3-phosphate dehydrogenase (GAPDH) functioned as the endogen control gene. The concentration of cDNA used as input for semiquantitative Real Time PCR (qPCR) differed from 50–100 ng. The qPCR volume was 13 µL, whereas 6.5 µL 2× mastermix (Applied Biosystems, Foster City, CA, USA), 2.5 µL primer mix (1.25 µM) and 0.5 µL probe (0.5 µM).

miRNA analyses were performed after a method published by Balcells et al. [64]. Briefly, miRNA is first polyadenylated and then reverse transcribed with a special primer (RT-primer). For qPCR analyses, two specific primers for each miRNA were designed using a software tool from Busk [65]. For miRNA analysis from tissue, an amount of 500 ng, for plasma of 50 ng total RNA containing miRNA, was reverse transcribed with 1 µM RT-primer (sequence shown in Table 1), 0.1 mM dNTP mix, 1 mM ATP, 100 U M-MLV reverse transcriptase (Promega), 1 U Poly-A-Polymerase (New England Biolabs, Frankfurt am Main, Germany), and 1 µL of 10× poly(A)polymerase buffer (New England Biolabs). The volume was filled up to 10 µL and incubated for 60 min at 42 °C. 10 ng cDNA from tissue and 1 ng cDNA from plasma were used as input for qPCR with 2× Bright Green (Promega, Mannheim, Germany). SnU6RNA functioned as the endogen control for all miRNA analyses performed in brain tissue. In plasma, miR 103 was used as reference miRNA. The mRNA and miRNA expressions were analyzed with the ABI Prism^®^ 7500 detection system (Applied Biosystems, Foster City, CA, USA) and QuantStudio5^®^ (Thermo Fisher, Darmstadt, Germany) according to the 2^−ΔΔ*C*T^ method [66].

### 4.4. Statistical Analyses

Experiments were performed in seven or eight animals per group (*n*  =  7–8). Data were analyzed using GraphPad Prism 5 (GraphPad Software, La Jolla, CA, USA). Values are presented as means ± standard error of the mean (SEM). Comparisons among groups were made using the Mann-Whitney *U*-test. *p* Values of <0.05 was considered to be significant.

## 5. Conclusions

Dexmedetomidine attenuated LPS-induced neuroinflammation via reducing *IL1-β* and *TNF-α* expression in the hippocampus and cortex of adult rats. Moreover, the drug prevented upregulation of miR 124, 132, 134 and 155 after LPS application in both investigated brain regions.

## Figures and Tables

**Table 1:** Primer and probe sequences for messenger RNA (mRNA) and microRNA (miRNA) analyses. All primers and probes were synthesized from metabion. F: forward primer, R: reverse primer, P: probe, RT: reverse transcription.

*IL1-β* NM_031512	F	aacaaaaatgcctcgtgctgtct	*TNF-α* NM_012675	F	tcgagtgacaagcccgtagc
	R	tgttggcttatgttctgtccattg		R	ctcagccactccagctgctc
	P	6-fam-acccatgtgagctga aagctctccacc-tamra		P	6-fam-cgtcgtagcaaacca ccaagcaga-tamra
GAPDH NM_017008	F	gatgctggtgctgagtatgtcgt	RT-primer		caggtccagttttttttttttttt
	R	tcaggtgagccccagcct			
	P	6-fam-tctactggcgtcttc accaccatggaga-tamra			
miR 103 MIMAT0000824	F	gcagagcagcattgtacag	miR 134 MIMAT0000840	F	gcagtgtgactggttgac
	R	ggtccagtttttttttttttttcatag		R	cagtttttttttttttttcccctct
miR 124 MIMAT0004728	F	gcagcgtgttcacagc	miR 155 MIMAT0030409	F	cgcagttaatgctaattgtgatag
	R	tccagtttttttttttttttcaaggt		R	aggtccagtttttttttttttttacc
miR 132 MIMAT0008381	F	gcagtaacagtctacagcca	snU6RNA NR_004394	F	atacagagaagattagcatggcc
	R	gtccagtttttttttttttttcgac		R	cgaatttgcgtgtcatccttg

## Abbreviations

α-7-nAChR	α-7 Nicotinic acetylcholine receptor
ALS	Amyotrophic Lateral Sclerosis
cDNA	Complementary DNA
CNS	Central Nervous System
DEX	Dexmedetomidine
EDTA	Ethylene-Diamine-Tetra-Acetic acid
GAPDH	Glyceraldehyde-3-Phosphate Dehydrogenase
i.p.	Intraperitoneal
IL1	Interleukin 1
LPS	Lipopolysaccharide
MAPK	Mitogen-Activated Protein Kinase
MCI	Mild Cognitive Impairment
miRNA	MicroRNA
M-MLV	Moloney Murine Leukemia Virus
mRNA	messengerRNA
MS	Multiple Sclerosis
MyD88	Myeloid Differentiation Factor 88
NF-κB	Nuclear factor kappa-light-chain-enhancer of activated B cells
PCR	Polymerase Chain Reaction
qPCR	Quantitative Polymerase Chain Reaction
SHIP1	Phosphatidylinositol-3,4,5-trisphosphate 5-phosphatase 1
SOCS1	Suppressor of cytokine signaling 1
TLR	Toll Like Receptor
TNF-α	Tumor necrosis factor-α
